# Evaluation of Electric Scooter Head and Neck Injuries in Paris, 2017-2019

**DOI:** 10.1001/jamanetworkopen.2020.26698

**Published:** 2020-11-20

**Authors:** Quentin Hennocq, Thomas Schouman, Roman Hossein Khonsari, Nicolas Sigaux, Vianney Descroix, Chloé Bertolus, Jean-Philippe Foy

**Affiliations:** 1 Department of Medicine, Sorbonne Université, Paris, France; 2 Department of Maxillo-Facial Surgery, Assistance Publique des Hôpitaux de Paris, Paris, Groupe Hospitalier Pitié-Salpêtrière Charles Foix, Paris, France; 3 Assistance Publique Hôpitaux de Paris, Hôpital Necker-Enfants Malades, Service de Chirurgie Maxillo-faciale et Chirurgie Plastique; Université Paris. Descartes, Université de Paris, Paris, France; 4 Department of Maxillofacial Surgery, Hospices Civils de Lyon, Hôpital Lyon Sud, Université Claude Bernard Lyon 1, Lyon, France; 5 Department of Odontology, Assistance Publique des Hôpitaux de Paris, Paris, Groupe Hospitalier Pitié-Salpêtrière Charles Foix, Paris, France

## Abstract

This case series study evaluates head and neck injuries caused by electric scooters in Paris, 2017-2019, focusing on user behavior and injury types.

## Introduction

Recent studies have highlighted the dramatic increase in injuries and admissions associated with electric scooter (e-scooter) use in several countries.^1,2,3,4,5,6^ Although self-service e-scooters have been available in Paris since June 22, 2018, in our department we have recently observed a trend toward an increase in severe head and neck injuries caused by the use of e-scooters.

The aim of this case series study was to assess the epidemiology of head and neck and dental trauma related to e-scooter use in Paris, focusing on user behavior and injury type.

## Methods

We performed an observational retrospective and prospective case series study between January 1, 2017, and October 31, 2019. The local ethics committee, Comité Scientifique et Ethique de l’Entrepôt de Données de Santé, approved the study, and all patients gave verbal informed consent. After study approval, we retrospectively requested 2 facial trauma centers to search their databases to retrieve all electronic medical records including the keyword *e-scooter* for the period between January 1, 2017, and June 30, 2019. Then one of us (Q.H.) manually screened medical records to identify patients treated for e-scooter–related head and neck trauma.

Data on age, sex, site, type (fractures, soft-tissue wounds, and dental lesions) of injured patients were collected from medical records by one of us (Q.H.). Mandibular fractures were described as unifocal (1 mandibular site) or plurifocal (>1 mandibular site).

We constructed a questionnaire including 9 items related to the circumstances of the accidents: alcohol or drug consumption (items 1 and 2), high-speed driving (3), driving on sidewalks (4), tandem riding (5), riding without a helmet (6), level of experience (>10 uses) with riding an e-scooter (7), experience with motorcycle driving (8), and possession of a driver’s license (9). A rider’s behavior was defined as risky if 1 of the 6 first items was answered as positive. From July 1 to October 31, 2019, items were prospectively collected by the surgeon who initially managed the patient’s care. Before July 1, 2019, items were retrospectively collected during a telephone call from one of us (Q. H.). This study followed the Strengthening the Reporting of Observational Studies in Epidemiology (STROBE) reporting guideline for observational studies.

## Results

Of the total of 125 patients, 49 (39%) were included prospectively and 76 (61%) were included retrospectively for facial (n = 92) or dental (n = 33) trauma associated with use of an e-scooter. An increase in such injuries was observed over the study period (Figure). The questionnaire was completed by 43 of 49 patients (88%) and 42 of 76 patients (55%) during the prospective and retrospective parts of the study, respectively. Only 11 injuries (9%) occurred over the time span of the study before the introduction of e-scooter self-service providers. The mean (SD) patient age was 32.5 (12.7) years, and 46 patients (37%) were women (Table). Two patients (2%) were pedestrians struck by e-scooters, including the oldest member of the study cohort (95 years). Only 10 patients (12%) used a helmet while riding. High-speed and sidewalk riding were noted in 44 of 87 patients (51%) and 26 of 84 patients (31%), respectively, during the 2 parts of the study. Alcohol consumption was reported in 45 of 92 cases (49%). Overall, a risky behavior was observed in 80 of 92 cases (87%).

**Figure.**
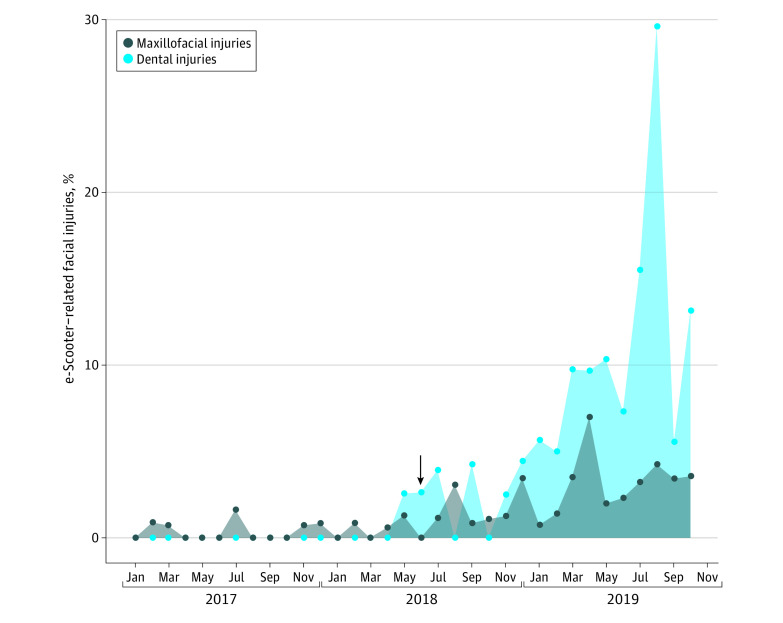
Time Trends of Electric Scooter (e-Scooter)–Related Head and Neck Injuries The arrow indicates the introduction of self-service e-scooters in Paris, France, on June 22, 2018.

**Table.** Patient Characteristics and Description of Facial Injuries Associated With Electric Scooters (e-Scooters)

Characteristic	No. (%)^a^
**Patients**	
Female	46 (37)
Age, y	
Mean (SD)	32.5 (12.7)
Median (range)	30 (10-95)
<18	3 (2.4)
Pedestrian hit by an e-scooter	2 (1.6)
Alcohol consumption	45 (49)
Drug consumption	5 (6)
Speed at the time of the accident^b^	
Low or medium	43 (49)
High	44 (51)
Driving on the sidewalk at the time of the accident	26 (31)
Tandem riding	12 (14)
User level (>10 e-scooter uses before the accident)	51 (60)
Previous experience with motorcycle driving	23 (27)
Driver’s license	51 (60)
Helmet use	10 (12)
**Facial injuries**	
Mandibular fractures^c^	66 (55)
No. of injured patients	36 (47)
Type	
Unifocal	4 (72)
Plurifocal	22 (28)
Site	
Dentulous portion	21 (32)
Condylar process	45 (68)
Midface fractures	53 (45)
No. of injured patients	43 (56)
Site	
Nasal bones	20 (26)
Zygoma	13 (17)
Occlusofacial fracture (Le Fort)	4 (5)
Orbital fracture	7 (9)
Craniofacial fractures	
No. of injured patients	7 (9)
Site	
Fronto-orbito-ethmoidal fracture	1 (1)
Frontal sinus	7 (9)
Facial wounds	90
No. of injured patients	78 (62)
Site	
Supraorbital rim/forehead	16 (18)
Nose	10 (11)
Cheek	3 (3)
Lips	25 (28)
Tongue	1 (1)
Chin	36 (40)
Dental trauma	115
No. of injured patients	55 (44)
Type	
Crown fractures	69 (60)
Dental dislocation	40 (35)
Alveolodental fractures	6 (5)
Site	
Incisors	92 (80)
Other teeth	23 20)

^a^ Denominators to calculate percentages may vary according to available data retrieved from the questionnaire as well as the medical records.

^b^ Patients were divided into 3 groups according to the speed at time of the accident: low (<10 kph), medium (10-20 kph), and high (>20 kph).

^c^ Each mandibular site of fracture in a patient was recorded as 1 fracture.

Two intracranial lesions, including a subdural hematoma and a subarachnoid hemorrhage, were reported. A surgical procedure with the patient under general anesthesia was performed in 36 of 77 patients (47%) with facial fractures.

## Discussion

Although trauma caused by e-scooters is poorly described in the literature, recently there has been an increased number of scientific publications on e-scooter–related injuries.^1,2,3,4,5,6^ Most published articles were retrospective observational studies from the United States.

Although the retrospective part of our study could bias the interpretation of our results, we noted that 87% of traumatic injuries were associated with risky behavior. e-Scooter companies explicitly do not recommend consumption of drugs or alcohol while riding. We believe that further information campaigns could help raise awareness of risky behavior during e-scooter riding to prevent e-scooter accidents and decrease related hospital admissions in emergency departments.

In summary, e-scooter accidents are increasing in the streets of Paris. Engaging all public authorities in the prevention of e-scooter–related accidents is urgently needed.
